# Beyond the Status Quo: Density Functional Tight Binding and Neural Network Potentials as a Versatile Simulation Strategy to Characterize Host–Guest Interactions in Metal- and Covalent Organic Frameworks

**DOI:** 10.1021/acs.jpclett.3c00941

**Published:** 2023-06-23

**Authors:** Thomas S. Hofer, Risnita Vicky Listyarini, Emir Hajdarevic, Lukas Maier, Felix R. S. Purtscher, Jakob Gamper, Friedrich Hanser

**Affiliations:** † Institute of General, Inorganic and Theoretical Chemistry, Center for Chemistry and Biomedicine, University of Innsbruck, Innrain 80-82, A-6020 Innsbruck, Austria; ‡ Institute of Electrical and Biomedical Engineering, UMIT Tirol, Eduard-Wallnöfer-Zentrum 1, A-6060 Hall in Tirol, Austria

## Abstract

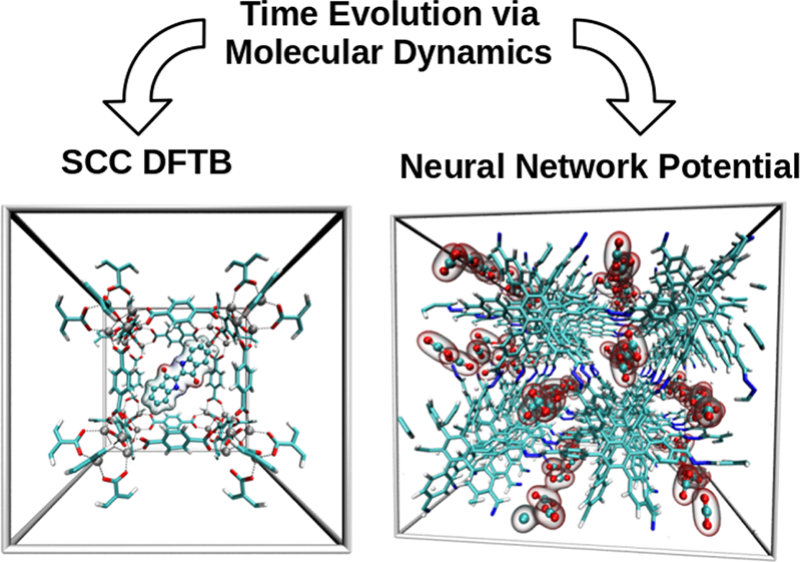

In recent years, research focused on synthesis, characterization, and application of metal–organic frameworks (MOFs) has attracted increased interest, from both an experimental as well as a theoretical perspective. Self-consistent charge density functional tight binding (SCC DFTB) in conjunction with a suitable constrained molecular dynamics (MD) simulation protocol provides a versatile and flexible platform for the study of pristine MOFs as well as guest@MOF systems. Although being a semi-empirical quantum mechanical method, SCC DFTB inherently accounts for polarization and many-body contributions, which may become a limiting factor in purely force field-based simulation studies. A number of examples such as CO_2_, indigo, and drug molecules embedded in various MOF hosts are discussed to highlight the capabilities of the presented simulation approach. Furthermore, a promising extension of the outlined simulation strategy toward the treatment of covalent organic frameworks utilizing state-of-the-art neural network potentials providing a description at DFT accuracy and force field cost is outlined.

As a consequence of their unique and versatile properties, nanoporous materials (NMs) enjoy widespread applications in science and technology.^1^ Naturally occurring NMs such as zeolithes and activated carbon display high porosities associated with large surface areas and are employed with great success as sorbents, catalysts, and ion exchangers.^2,3^ Moreover, promising applications as gas storage media^4^ and solid-state battery materials^5^ have been discussed. Although different classes of naturally occurring nanoporous compounds have been identified in the past, the emergence of supramolecular chemistry in the 1960s paved the way toward an entire new class of functional materials referred to as metal–organic frameworks (MOFs).^6,7^ While often considered as a separate class from supramolecular coordination complexes, both structure types share a common building block comprised of organic molecules linking neighboring metal-containing coordination sites^8,9^ referred to as secondary building units (SBUs). Later, in the mid 2000s, research into nanoporous materials was extended to purely organic building blocks by replacing the metal-containing SBU with a covalently bound central moiety.^10,11^ Similarly, as in the case of MOFs, research focused on these covalent-organic frameworks (COFs) enjoys rapidly increasing interest, with potential applications touching virtually every area of science and technology.

At present, MOFs and COFs represent two of the most rapidly growing classes of nanomaterials. This is due to the near-infinite possibilities when combining a specific center moiety with organic linker molecules. Today, potential applications of these compounds by far exceed those of natural NMs, extending *inter alia* into areas such as (semi)conducting and optical functionalized materials^12−14^ (e.g., light harvesters, photoswitches, *etc.*), molecular sensing,^15^ as well as drug delivery.^16^

Considering the rapid success in the development of novel MOF/COF compounds, on the one hand, and the nearly limitless possibilities in combining different central and linking units, efficient strategies to assess the properties of potential candidate structures at the atomic level prior to their synthesis in the lab are of particular interest. Theoretical approaches aimed at the description of chemical systems have made impressive progress over the past decades. Parametrized empirical potential models (commonly referred to as molecular mechanics, MM, or force fields, FF) enable the study of virtually every class of chemical system and have been applied with great success in the study of MOF materials^17−19^ as well. Though highly efficient in terms of execution times, these approaches are oftentimes limited in the achievable accuracy, especially when pronounced polarization, charge transfer, or/and many-body contributions are relevant for the treatment of the investigated compound. Indeed, a number of articles have discussed these shortcomings in the description of MOF systems: while FF-based approaches are capable of providing accurate descriptions of pristine MOFs,^18^ the treatment of open metal sites^20^ and interactions associated with guest molecules embedded in the MOF host were reported to oftentimes suffer from the limited accuracy in the force field description.^21−24^

In contrast, the above-mentioned contributions are inherently taken into account if quantum mechanical (QM)^25^ calculation methods such as density functional theory (DFT)^26^ are employed. However, this advantage comes at the cost of a massively increased computational demand, which is further amplified by an unfavorable scaling upon increase in system size. The latter disadvantage of QM approaches highlights the challenging nature of applying QM-based calculation methods in investigations of MOF systems. Due to their supramolecular nature, the number of atoms and, consequently, the number of electrons are comparably high. Although DFT methods have been successfully applied to describe MOF systems in the past, these calculations often consider just a high symmetry configuration subject to an energy minimization to identify the respective optimized structure. In many cases such a description is sufficient to derive a variety of properties of the target system (e.g., conformational energy differences, vibrational frequencies, *etc.*). However, it can be shown based on arguments taken from statistical thermodynamics^27^ that a description based on a single minimum structure corresponds in a strict sense to 0 K conditions.

A large number of phenomena in the chemical sciences cannot be adequately described by such a simplified approach. In these cases a representation based on a thermodynamic ensemble,^27^ i.e., a collection of structures weighted according to their probability of occurrence, is required to describe the system at a specific state point, i.e., at nonzero temperatures and pressures. Both the Monte Carlo (MC) and molecular dynamics (MD) simulation approaches enable the accumulation of configurations associated with such an ensemble.^27^ However, since a very large number of configurations is required (typically in the range of tens of thousands up to several hundred million), quantum chemical approaches prove too demanding to achieve an adequately converged ensemble of typical MOF and COF systems.

However, if the computational complexity of the QM calculation can be reduced, e.g. by utilizing approximate DFT methods based on the generalized-gradient approximation,^26^ the application of a QM-based description of the energy and forces can indeed be combined with an MD simulation protocol. A particularly successful strategy to achieve such a compromise between accuracy of results and computational effort is the application of semi-empirical calculation methods.^28^ The latter comprise a highly efficient class of computational approaches that are initially derived from QM-based methodologies but employ large sets of empirical parameters to reduce the computational demand. The self-consistent charge density functional tight binding (SCC DFTB) approach,^29^ based on a Taylor expansion of the Kohn–Sham energy of DFT methods with respect to variations in the equilibrium density, proved to be a versatile and surprisingly robust approach for the treatment of metal–organic frameworks and associated guest@host systems.^30,31^

DFTB approaches are based on highly simplified tight binding Hamiltonians that are parametrized against a suitable DFT reference.^29^ Common approximations in DFTB methods involve the restriction of the description to valence electrons and the assumption that contributions to the Hamiltonian elements *H*_*μν*_ are solely based on the pair-distance and angular momenta of the interacting orbitals (e.g., ss^σ^, sp^σ^, pp^σ^, pp^π^, ..., dd^δ^, ...). The SCC DFTB energy expression is given as
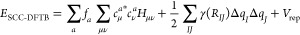
1with the indices μ and ν being associated to the orbitals *n* in the systems, while indices *I* and *J* refer to the respective nuclei *N*. The index *a* refers to all occupied states *n*, with the respective occupation *f*_*a*_ being typically expressed via the Fermi function^26^ including a factor of 2 to account for double occupancy. The function γ(*R*_*IJ*_) corresponds to a damped Coulomb-type interaction based on the respective pair distance *R*_*IJ*_, which approaches the well-known *R*^–1^ dependence at large distances. In the case *I* = *J*, the respective Hubbard *U*_*I*_ parameter, accounting for charge transfer to or from a particular atom, is applied. It can be shown that *U*_*I*_ is directly related to the concept of chemical hardness η *via*
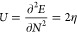
2

Finally, all contributions not included in the Hamiltonian elements and the charge transfer terms can be combined in good approximation into pairwise atomic contributions, which are commonly referred to as repulsive potential (*V*_rep_).

This level referred to as DFTB of second order (DFTB2) solves the electronic structure problem iteratively by adjusting the respective atomic populations expressed *via* the linear coefficients *c*_μ_^*a*^ (*a* ∈ *n*) and the associated atomic partial charges *Δq*_*I*_ (*I* ∈ *N*) until a minimum in the energy is achieved. Since typically the parent DFT methods such as PBE require empirical correction terms to improve the description of dispersive interactions (such as the Grimme D3 correction^32^), similar corrections are applied in DFTB-based studies as well.

In order to enhance the description of the target systems, a third-order expression has been introduced,^33^ requiring also the so-called Hubbard derivatives to represent the charge dependence of the chemical hardness of the individual atoms. A highly successful variant of this DFTB3 level is the 3ob parameter set^34^ introduced with a focus on organic and biologically relevant systems. While this implies applications to (bio)organic molecules including *inter alia* QM/MM-type simulations of peptides and proteins,^35^ the functional groups inherent to a large number of metal–organic frameworks display a high similarity to those encountered in metal-binding sites of biomacromolecules. For instance, the often encountered coordination of terephthalate (i.e., benzenedicarboxylate, BDC^2–^) or substituted immidazolate residues to metal ions such as Mg^2+^, Zn^2+^, or Cu^2+^ are comparable to the fixation of these ions in biological systems *via* aspartate, glutamate, and histidine residues.

Considering these similarities, it is not surprising that the SCC DFTB/3ob level proved perfectly adequate to accurately model the properties of a variety of MOF systems as well. The latter is not only true in terms of energy minimizations routinely carried out to identify minimum configurations on the potential energy surface.^30,31^ Indeed, applications of this level of theory in MD simulations were highly successful in describing MOFs and guest@MOF systems.^36−39^ In a recent publication,^39^ the capabilities of various DFTB-based methods to describe a number of pristine MOF systems have been investigated, demonstrating that the SCC DFTB/3ob/D3 level provided a highly suitable description when comparing the average lattice constants, powder X-ray diffraction (XRD) patterns, and pair distribution functions with experimental reference data. Several similar MD simulations of azobenzene photoswitches embedded in DMOF-1 were able to directly link the atomic structure resulting from the host–guest interaction to the experimental observations in the photoswitching efficiency.^37,38^ To the best of our knowledge, the first MD study of an MOF system to employ a DFTB description was conducted by Li et al. investigating water adsorption in MOF-74.^36^ Despite employing only DFTB of second order and executing the MD simulations for short sampling times of 30–50 ps, a comparison of the calculated adsorption energies with data obtained from DFT calculations employing the vdw-DF functional has been reported, “validating DFTB suitable to investigate absorption properties, where ab initio quantum chemistry calculations are prohibitive”.^36^

An elegant and efficient approach to lower the computational demand associated with molecular dynamics simulations is the application of an increased MD time step in the range of 1.0–2.0 fs. This can be achieved by keeping the vibrational degrees of freedom associated with H-containing bonds frozen by introducing so-called holonomic constraints.^27^ This approach considering selected bonds as rigid is widely employed in FF-based simulations of biomacromolecular systems. However, the application of constraint algorithms such as the SHAKE and RATTLE procedures^27^ is not rooted in the application of force field approaches but can be interpreted as a partial integration of the associated partition function.^27^ What makes this approach challenging in the context of QM-based MD simulation is the fact that the target bond lengths of the individual constraints must be provided as input. The latter can be obtained by executing MD simulations at the target conditions employing shorter timesteps in the range of 0.1–0.5 fs, thereby enabling full flexibility of all bonds in the systems. Based on the resulting simulation data the ensemble averages of all X–H bonds (with X typically being C, O, and N) can be determined, which then act as target distances for the associated constraint procedure. Since the C–H bonds of aliphatic and aromatic moieties are largely independent of the applied temperature and pressure, the determined reference bond lengths can be used to a very good approximation in simulations over a wide temperature and pressure range. However, for N–H and O–H bonds that are prone to proton transfer reactions, the transferability of the determined average bond distances should be carefully considered.

While the associated computational demand makes these SCC DFTB MD simulations comparably demanding when compared to pairwise-additive force fields (even if rigid bonds are employed to increase the MD time step), these approaches provide manifold advantages in the treatment of MOF systems. For instance, it could be shown that a much better agreement between experimental and theoretically derived X-ray diffraction patterns is achieved when compared to XRD data obtained from the respective minimum configuration corresponding to 0 K conditions.^39^ Since the time evolution of the target system resulting from an MD study yields a large number of individual configurations, ensemble-averaged diffractograms can be generated by overlaying hundreds to several thousand individual XRD patterns calculated at regular intervals over the MD trajectory. Because the system is represented at a well-defined state point, the accuracy of the theoretical approach can be directly evaluated against the experimental reference determined under similar conditions. That way, the influence of additional factors, such as the thermal expansion of the unit cell parameters, is inherently taken into account.

This is demonstrated in the case of MOF-5 and ZIF-8, two well-studied and highly prominent MOF systems treated at the SCC DFTB/3ob/D3 level (see Figure 1). Both powder X-ray diffraction (PXRD) patterns obtained (i) from energy minimization and (ii) *via* averaging over the MD trajectory show very good agreement with the experimental reference. However, close inspection of the MD-averaged PXRD patterns shown in Figure 1c,d reveal a slight shift toward the experimental results in both cases. While a shift to larger angles is visible in the case of MOF-5, a decrease in the angles is observed for ZIF-8. This difference is the result of the thermal expansion of the individual MOFs at elevated temperatures, which is reported to be negative in the case of MOF-5, while ZIF-8 is known to display a positive thermal expansion. This finding prompted the investigation of the associated thermal expansion coefficient by monitoring the average lattice constant *a* of the cubic unit cells at ambient pressure (1.013 bar) as a function of the simulation temperature shown in Figure 1e,f. It can be seen that despite the comparably long simulation times amounting to 250–300 ps for each temperature point, notable standard deviations in the lattice parameters are observed. This is a result of the comparably small number of atoms in the unit cell containing 276 and 424 atoms in the case of ZIF-8 and MOF-5, which on the other hand correspond to 888 and 1552 valence electrons, respectively. Nevertheless, the positive and negative thermal expansions of the individual MOFs are clearly visible.

**Figure 1:**
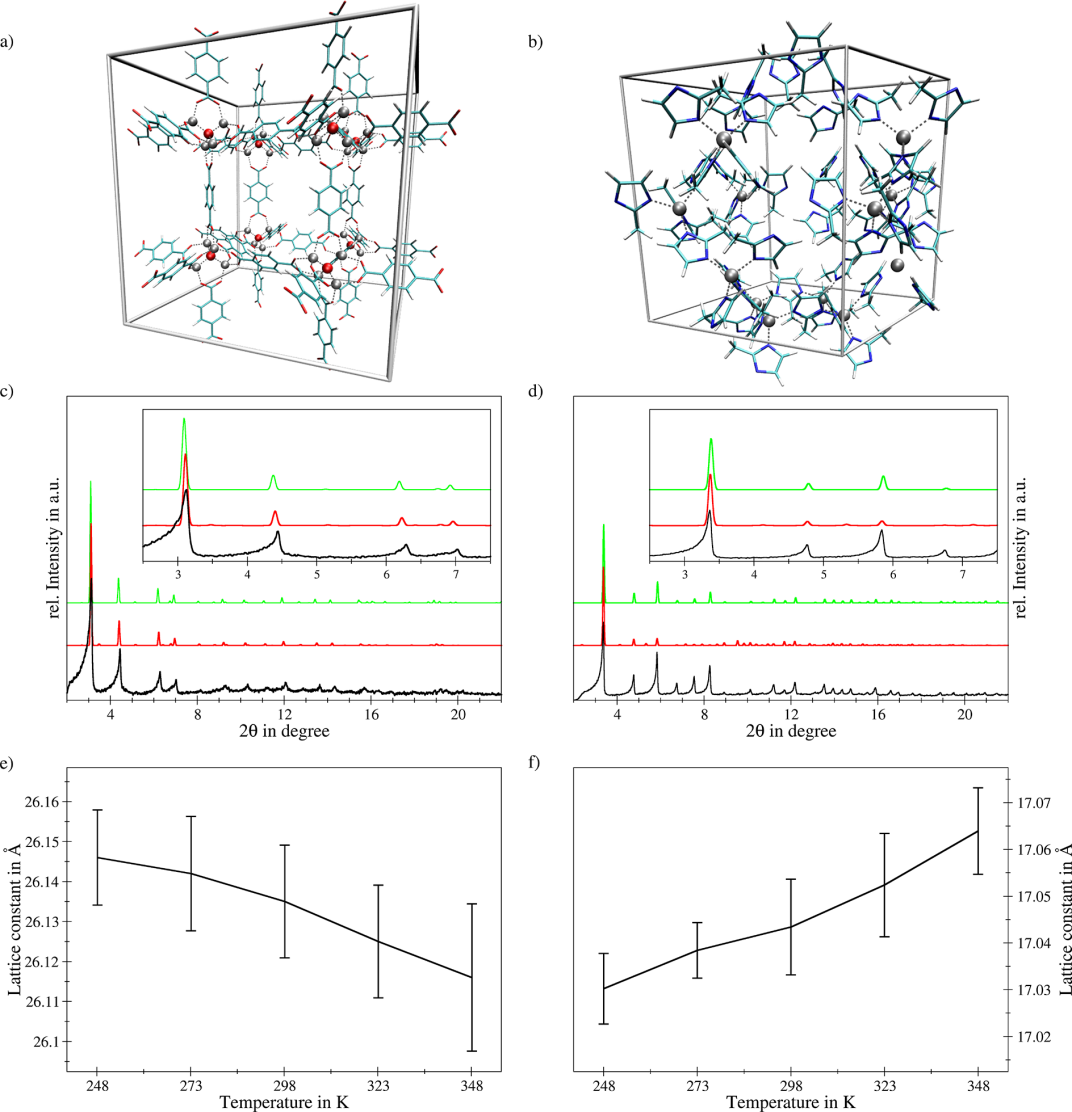
Unit cells of the simulation systems of (a) MOF-5 and (b) ZIF-8 employed in SCC DFTB/3ob/D3 MD simulations along with a comparison of (c and d) the associated PXRD patterns (Mo K_α_, λ = 0.709319 nm) obtained *via* MD simulations at 298 K (red) and energy minimization corresponding to 0 K conditions (green) with the respective experimental reference (black). (e and f) Temperature dependence of the associated lattice constants determined *via* SCC DFTB/3ob/D3 MD simulations at ambient pressure (1.013 bar) reflecting the respective negative and positive thermal expansion.

Based on the simulation results, a negative thermal expansion coefficient of −12.7 MK^–1^ has been determined for MOF-5, in very good agreement with experimental and calculated estimates reported in the range from −16 to −10 MK^–1^ and −26.2 to −5.3 MK^–1^, respectively (see the Supporting Information, Table S1). Similarly, a good estimate of 14.6 MK^–1^ has been determined in case of ZIF-8, which again compares very well with the experimental value reported in the range of 6.5 to 11.9 MK^–1^ (see Table S1). The approach can also easily be extended to more complex MOF systems as for instance the binary MOF UMCM-9 comprised of the same Zn_4_O^6+^ unit as found in MOF-5 that are connected by two different linkers, namely, 1,1′-biphenyl-4,4′-dicarboxylate and 2,6-naphthalenedicarboxylate (see Figure S1 and Table S1).

The fact that the application of the SCC DFTB/3ob/D3 level accurately represents the positive and negative thermal expansion of these well-known MOF systems is quite remarkable. In addition to also providing accurate estimates for PXRD patterns, it should be noted that the simulations directly confirm that the employed level of theory provides an adequate description of the neat MOF systems at elevated temperature and ambient pressure conditions. No tendency toward structural rearrangement such as a collapse of the pore structure is observed along these comparably long MD simulations.

Since the structural integrity of the host system is a crucial prerequisite for further studies, this finding provides the basis to evaluate the performance of DFTB in the treatment of more complex guest@MOF systems. While the latter have also been studied employing more efficient force field approaches, several articles discuss potential limitations in the description of the host–guest interactions mostly due to missing many-body contributions such as polarization.^20−24^ The latter are inherently taken into account in QM-based calculation methods, and while DFTB represents a highly parametrized semi-empirical approach, these effects can still be expected to be adequately considered at this level of theory as indicated by the very good agreement in the theoretical and experimental XRD patterns.

The interaction between carbon dioxide, a prominent greenhouse gas, and MOF-5 represents a well-studied model system. While energy minimizations might provide a straightforward route to investigate the associated host–guest interactions, the resulting minimum geometry is strongly dependent on the chosen initial structure. However, as a consequence of the supramolecular nature of MOF systems, several binding positions with different characteristics may be present within the same system. While being computationally more demanding, molecular dynamics simulations enable a probing of potential interaction sites at different temperatures, thereby providing also access to time-dependent properties, e.g., anharmonic vibrational spectra and diffusive properties.

Figure 2a displays the coordination motif of a CO_2_ molecule at a Zn_4_O^6+^ unit of MOF-5 observed repeatedly in an SCC DFTB MD simulation of a single guest molecule in the MOF host. Based on the simulation data, the average host–guest interaction energy *U*_int_ can be determined (see Figure 2b). While at first sight the large variation in the interaction energy of ±200 kJ mol^–1^ appears to be entirely out of scope when considering the interaction of a CO_2_ molecule with the MOF host, it should be noted that the time evolution of *U*_int_ still accounts for all geometrical changes in the host structure, including variations of the unit cell due to the constant pressure treatment. Especially the latter are known to often result in large variations in the instantaneous energy. However, as can be seen from the running average, these fluctuations cancel when evaluating the respective ensemble average ⟨*U*_int_⟩. Despite the large amplitudes in the oscillation of the instantaneous energy the average value of −13.0 kJ mol^–1^ determined over the second half of the simulation compares very well with experimental estimates^40^ for the heat of adsorption *ΔH*_ads_ reported as −15.1 and −14.9 kJ mol^–1^ and other theoretical estimates^40,41^ amounting to −9.3 and −14.0 kJ mol^–1^. This highly encouraging data prompted an analysis of the diffusive properties of CO_2_ embedded in the MOF structure. Since according to Einstein diffusion has to be analyzed *via* the mean square displacement (MSD) in the long-time limit,^42^ much longer sampling periods are required compared to the determination of structural properties such as the average lattice constants and XRD patterns. In this study, an equilibration period of ≥25 ps has been employed followed by 0.5 ns of sampling for each of the seven considered temperatures being equally spaced in the range from 248 to 398 K (increments of 25 K). The resulting MSD plots evaluated using a correlation length of 5 ps are depicted in Figure S2. For all considered temperatures, a near-ideal linear dependence of the MSD in the long-time limit is observed, which is well separated from the associated ballistic regime. The self-diffusion coefficient *D*_*s*_ of a single CO_2_ molecule embedded in MOF-5 determined at 298 K and 1.013 bar as 2.47 × 10^–8^ m^2^ s^–1^ compares very well to other theoretical results^43,44^ reported as 1.4 × 10^–8^ and 3.0 × 10^–8^ m^2^ s^–1^, respectively. Figure 2c displays the Arrhenius representation of *D*_*s*_ determined *via* SCC DFTB MD simulations for 7 evenly spaced temperatures in the range of 248 to 398 K. Although the activation energy of 4.3 kJ mol^–1^ determined for the entire temperature range (red line in Figure 2c) agrees very well with the value of 4.05 kJ mol^–1^ reported by Babarao and Jiang based on classical MD simulations,^43^ the dependence of ln(*D*_*s*_) with respect to the inverse temperature displays a notable deviation from the ideal linearity. Due to the (in general) exponential dependence of the diffusion coefficient with respect to temperature, the number of diffusive events observable within MD simulations is notably increased at elevated thermal conditions. For this reason, the estimation of the diffusion coefficient becomes less reliable at lower temperatures which may lead to a divergence from an ideal linear Arrhenian behavior.^45^ If only the four highest temperatures are considered in the linear fit (green line in Figure 2c) a higher value for *E*_*a*_ of 7.4 kJ mol^–1^ is obtained that agrees very well with the experimental estimate of 7.61 kJ mol^–1^.^46^

**Figure 2:**
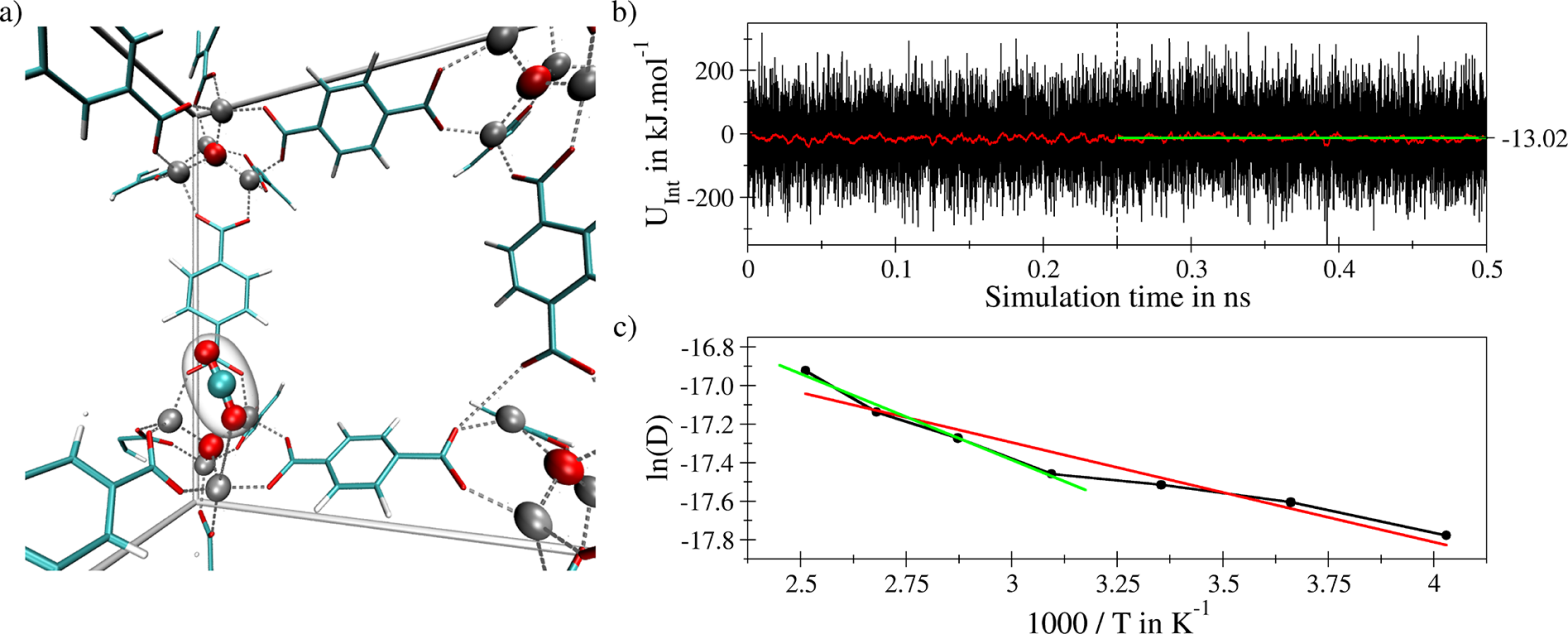
(a) Recurring interaction motif between a single CO_2_ molecule and a Zn_4_O^6+^ SBU of MOF-5 observed in the SCC DFTB/3ob/D3 MD simulation (snapshot taken from the interior of the unit cell). (b) Time series of the CO_2_-MOF-5 interaction energy (black) and associated running average based on a window size of 500 data points (red). Only the last 0.25 ns of the simulation trajectory have been considered to determine the associated average value (green). (c) Arrhenius depiction of the logarithmic self-diffusion coefficient (D in m^2^ s^–1^) of CO_2_ (black) with respective linear fits considering the entire set (red) and the four highest temperatures (green), respectively.

The accurate description of both the pristine MOFs as well as the archetypal CO_2_@MOF-5 system clearly demonstrates that the outlined SCC DFTB MD simulation strategy provides a flexible and versatile framework that can be easily generalized toward the treatment of different guest molecules focused on applications beyond gas storage. Recently, the outlined methodology has been applied to investigate the interaction between DMOF-1 and photoswitchable molecules of the azobenzene family in *E*- and *Z*-conformation.^37,38^ The highly regularized orientation inherent to MOF structures ensures a controlled environment, thereby enhancing the reversibility of the embedded photoswitches, which can be further enhanced by controlling the amount of loading. Potential future applications of these novel functional switch@MOF materials include *inter alia* sensing, controlled light harvesting/emission, controlled capture and release of various gases, smart MOF membranes, and data storage.^47−49^

DFTB-based MD simulations provide detailed insights into structural, dynamical, and thermodynamical properties of the host–guest complex. Figure 3 displays simulation results obtained for 2,2′-Bis(2,3-dihydro-3-oxoindolyliden), i.e. indigo, a highly prominent and well-investigated lead compound for the design of photoswitchable molecules, embedded in MOF-5. While highly mobile, the conformation displayed in the snapshot in Figure 3 represents a recurring motif observed repeatedly over the simulation trajectory. Comparing the average interaction energies obtained from the SCC DFTB MD simulation as −99.0 and −107.6 kJ mol^–1^ it can be concluded that the *Z*-conformer displays a stronger interaction with the host material. This trend can be explained by the difference in the dipole moment of the indigo molecule determined as 0.02 and 7.20 Debye in the case of *E*- and *Z*-indigo at the respective minimum configurations. These values determined *via* SCC DFTB/3ob/D3 calculations compare well with estimations obtained using the more demanding B3LYP/6-311G(d,p)/D3 and MP2/6-311G(d,p) levels as 7.0 × 10^–3^ and 8.0 × 10^–3^ Debye in the case of the *E*-isomer, while again high values of 7.0 and 6.2 Debye are found in the case of *Z*-indigo, respectively.

**Figure 3:**
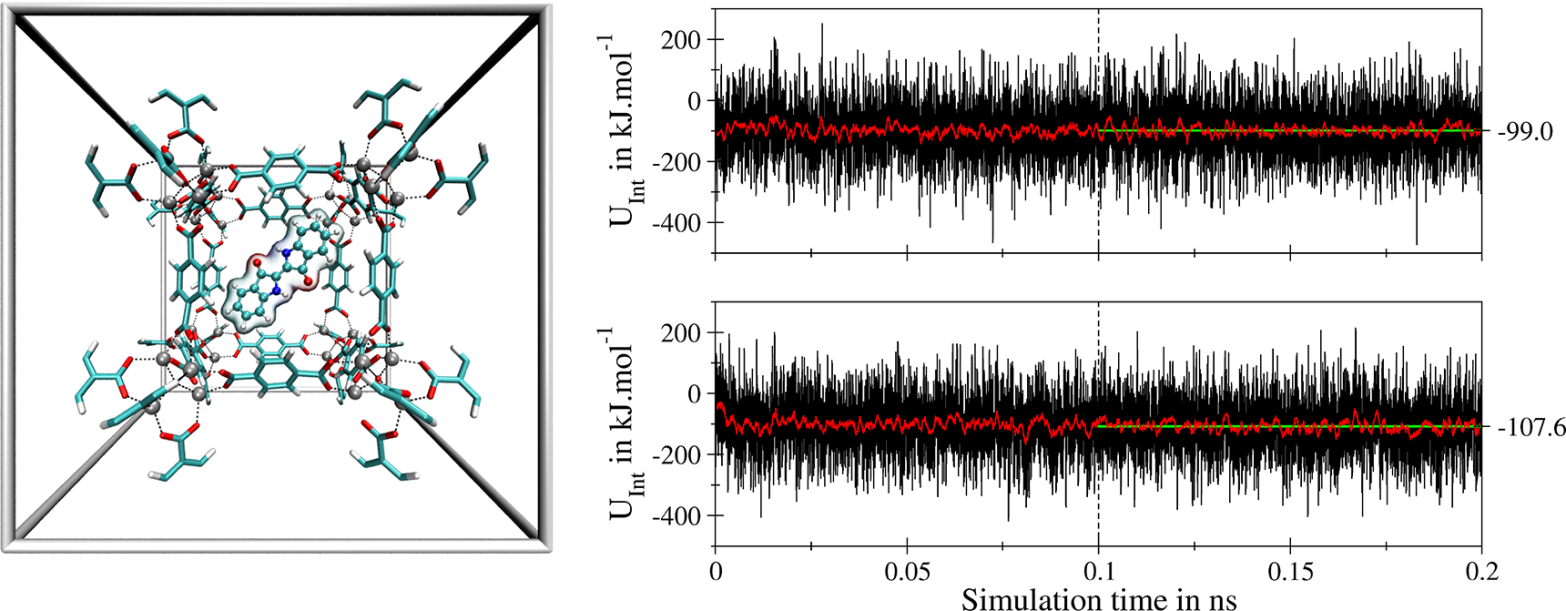
Left: Recurring interaction motif of a single *E*-indigo molecule with two Zn_4_O^6+^ SBUs of MOF-5 observed in the SCC DFTB/3ob/D3 MD simulation. Right: Time series of the interaction energy between the *E*- (top) and *Z*-form (bottom) of a single indigo molecule with the MOF-5 host (black) and associated running averages based on a window size of 500 data points (red). Only the last 0.1 ns of the simulation trajectory has been considered to determine the associated average values (green).

Following the successful examples in the treatment of guest@MOF systems presented above and in previous contributions,^37,38^ a large spectrum of potential applications can be envisioned employing the outlined SCC DFTB MD simulation protocol. An increasingly promising area of research is focused on the embedding of drug molecules in MOF hosts with the aim of enhancing drug delivery properties.^16^ Improved treatments in the regime of oncotherapy or alternative forms of application such as, for instance, the oral administration of insulin are just two examples of highly promising concepts in this area of research. Moreover, the prospect of improved drug delivery strategies can be expected to be an important contribution in the area of drug development: in recent decades a large number of active compounds failed to pass the high standards of clinical trials.^50^ It is expected that by embedding these promising compounds in a suitable MOF vector, the effective spectrum can be greatly enhanced, while at the same time side effects may be reduced to a large extent. Further applications of drug@MOF systems involve sensing applications such as antibody detection in wastewater.^51^

From the theoretical perspective, the treatment of drug molecules in an MOF host is virtually identical to the examples of CO_2_ and indigo embedded in MOF-5 discussed above. Figure 4 displays a selection of exemplary drug@MOF systems that have recently been investigated by employing the outlined SCC DFTB MD simulation strategy. The three target systems 5-fluorouracil@MOF-5, caffeine@ZIF-8, and nitrofurazone@[Zn_2_(T3CPPE)(H_2_O)_2_] were selected on the basis of previous experimental investigations demonstrating the successful loading of these MOFs with the respective guest molecule.^51−53^ In these examples, only the drug molecule has been considered as guest, whereas *in vivo* these compounds are exposed to an aqueous environment. In this context, ZIF-8 has been shown to exhibit a notable hydrothermal stability if it is subjected to a thermal treatment in an inert atmosphere, thereby partially carbonizing the outermost surface.^54^

**Figure 4:**
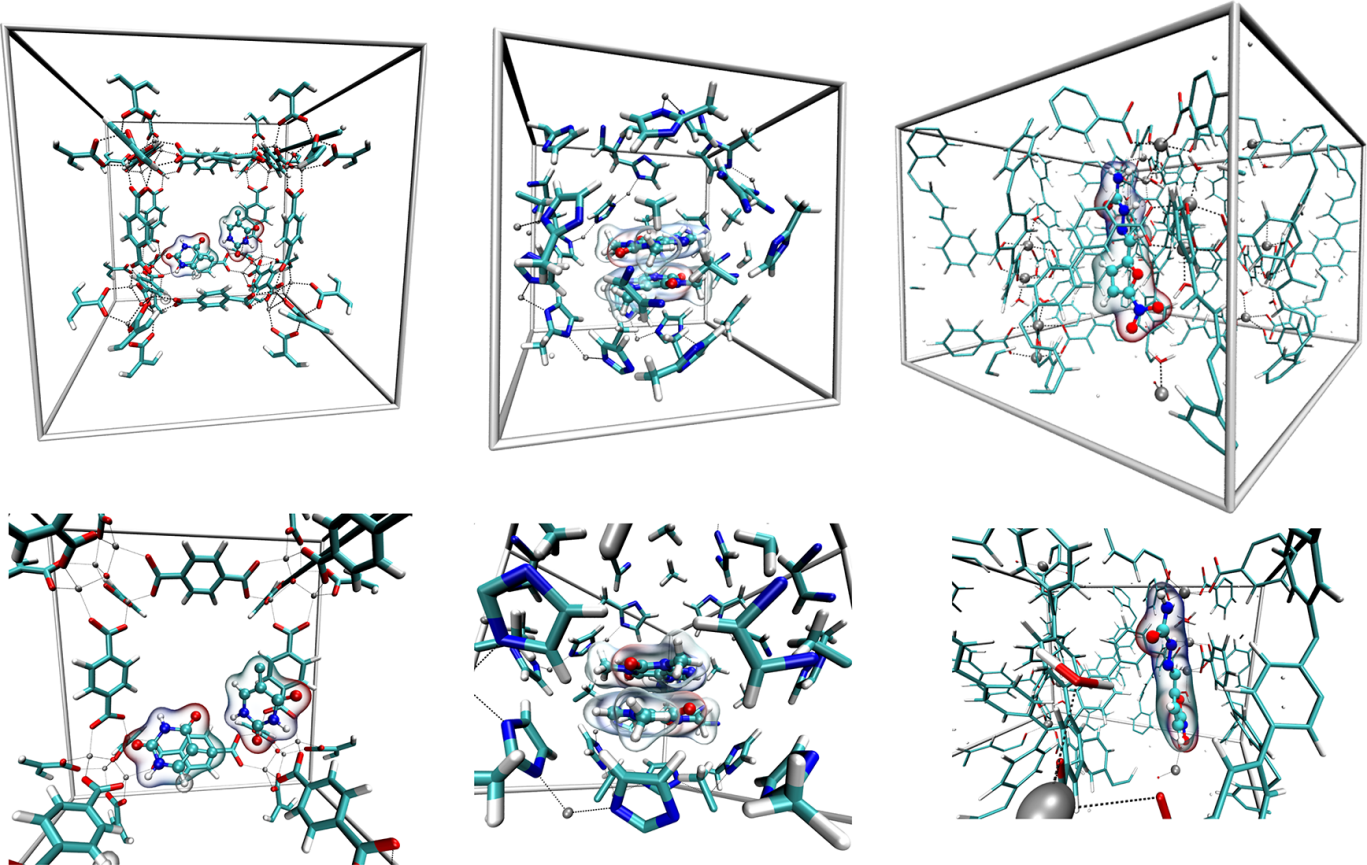
Snapshots taken from SCC DFTB MD simulations of three exemplary drug@MOF systems. Left: Two 5-fluorouracil molecules in MOF-5. Center: Two stacked caffeine molecules in ZIF-8. Right: Nitrofurazone embedded in the 2D luminescent metal–organic framework [Zn_2_(T3CPPE)(H_2_O)_2_].

Again, the SCC DFTB/3ob level of theory has been shown to provide an exceptionally good description of liquid water at ambient conditions if a suitable parametrization is employed.^55^ The outlined simulation strategy can thus be easily extended toward the study of drug@MOF systems and other functional molecules embedded in an MOF host under humid conditions by explicitly including water molecules in the simulation system. The application of constraint algorithms to the internal degrees of freedom of the H_2_O molecules is equally viable, thus enabling comparably long simulation times in the range of several hundred picoseconds up to a few nanoseconds. Moreover, the availability of GPU-accelerated DFTB routines can be expected to enhance the capabilities of this simulation approach even further, as already observed in the regime of FF-based computational approaches.

In addition to the above considerations, the astonishing progress in machine learning algorithms observed in the past decade also opens new avenues for the study of nanoporous compounds. In particular neural network potentials (NNP) such as ANAKIN-ME (Accurate NeurAl networK engINe for Molecular Energies, ANI)^56^ have been reported to provide energies and forces of target systems at DFT accuracy and force field cost. One astonishing feature of the ANI NNP is the accurate description of molecular systems that are substantially larger than those employed in the respective training set. While being at present limited to only a small number of elements (the second generation of the NNP ANI-2x^56^ considers C, H, N, O, S, F, and Cl), it provides direct access to a large number of covalent organic frameworks. Although appearing at first glance to be only partially related to the outlined SCC DFTB treatment of metal–organic frameworks, the combination of an NNP capable of describing COF systems with an adequate simulation protocol follows the same rationale: Also in this case, constrained MD algorithms keeping individual bonds rigid can be applied, with the respective equilibrium distances being determined *via* short, unconstrained MD runs.

Although it has been recommended to consider applications of the ANI-2x NNP to crystalline organic systems with caution, a recent study^57^ of CO_2_ diffusion in the COF systems HEX-COF1 and 3D-HNU-5 (see Figure 5) has demonstrated that the application of ANI-2x within a constrained molecular dynamics framework is capable of representing the pristine COF systems as well as the CO_2_-COF interactions with high accuracy. By analysis of the diffusion coefficients of the guest molecules as a function of loading, maxima in the associated activation energies could be identified for the different host materials. These maxima were found at loadings that coincide well with the experimentally determined maximum CO_2_ adsorption capacities, again indicating an exceptional storage capacity for these compounds.^57^

**Figure 5:**
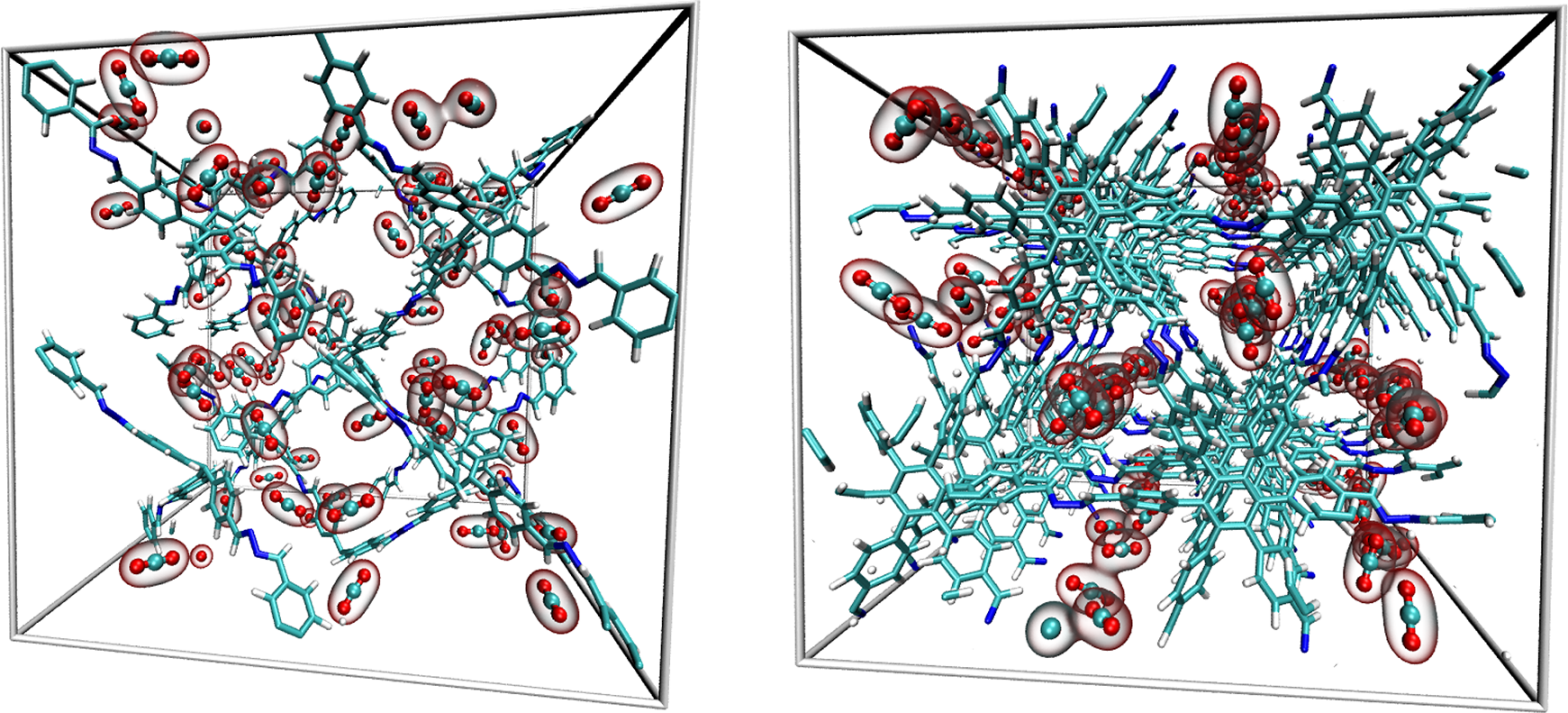
Snapshots taken from ANI-2x MD simulations of 64 CO_2_ molecules embedded in the three- and two-dimensional azine-linked COFs 3D-HNU-5 (left) and HEX-COF1 (right).

The studied target systems presented in this work provide clear evidence that modern computational approaches combining the capabilities of semi-empirical DFTB methods and neural network potentials with established constrained molecular dynamics frameworks provide access to a broad range of structural, dynamical, and thermodynamical properties of complex guest@host systems. Although the computational demand of the discussed SCC DFTB/3ob/D3 MD simulations is orders of magnitude higher compared to the application of pairwise additive force fields, the execution times are well within the manageable range. For instance, the average calculation time of a single energy and force evaluation of the systems presented in this study is found in the range of 7–12 s if executed in parallel using 4–12 computing cores. Thus, calculations reaching a total simulation time of 110 ps (10 ps of equilibration plus 100 ps of sampling corresponding to 5 000 and 50 000 MD steps) require net computing times of about 4–8 days. In case longer simulation times have to be achieved (e.g., to determine the diffusion coefficient of CO_2_@MOF-5), a minimum simulation time of 0.5 ns has to be reached, resulting in execution times of approximately 3 weeks for each of the investigated temperatures if executed on three Intel Xeon Platinum 8260 Quad-Core processors (i.e., 12 cores in total) using DFTBPLUS v21.2. Especially when considering current challenges such as climate change, the search for advanced energy technologies, and improvements in drug delivery, the continuous progress in computational methods can be expected to become increasingly important to complement, aid, and lead experimental investigations in the near future.

## Supporting Information Available

Methods, analysis, thermal expansion coefficients of MOF systems, mean square displacement of CO_2_@MOF-5 (PDF)

## References

[ref1] Lu G. Q.; Zhao X. S. Nanoporous Materials: Science and Engineering; Imperial College Press/World Scientific Publishing: London, 2004.

[ref2] Li Y.; Yu J. Emerging applications of zeolites in catalysis, separation and host–guest assembly. Nat. Rev. Mater. 2021, 6, 1156–1174. 10.1038/s41578-021-00347-3.

[ref3] Abd A. A.; Othman M. R.; Kim J. A review on application of activated carbons for carbon dioxide capture preparation, and surface modification for further improvement. Environ. Sci. Pollut. Res. 2021, 28, 43329–43364. 10.1007/s11356-021-15121-9. 34189695

[ref4] Boateng E.; Chen A. Recent advances in nanomaterial-based solid-state hydrogen storage. Materials Today Advances 2020, 6, 100022 10.1016/j.mtadv.2019.100022.

[ref5] Yue J.; Wang S.; Guo Y. Nanostructures and Nanomaterials for Batteries; Springer: Singapore, 2019; pp 215–263.

[ref6] Yuan S.; Feng L.; Wang K.; Pang J.; Bosch M.; Lollar C.; Sun Y.; Qin J.; Yang X.; Zhang P.; Wang Q.; Zou L.; Zhang Y.; Zhang L.; Fang Y.; Li J.; Zhou H. Stable Metal–Organic Frameworks: Design, Synthesis, and Applications. Adv. Mater. 2018, 30, 1704303 10.1002/adma.201704303. 29430732

[ref7] Safaei M.; Foroughi M. M.; Ebrahimpoor N.; Jahani S.; Omidi A.; Khatami M. A review on metal-organic frameworks: Synthesis and applications. Trends Analyt. Chem. 2019, 118, 401–425. 10.1016/j.trac.2019.06.007.

[ref8] Meek S. T.; Greathouse J. A.; Allendorf M. D. Metal-Organic Frameworks: A Rapidly Growing Class of Versatile Nanoporous Materials. Adv. Mater. 2011, 23, 249–267. 10.1002/adma.201002854. 20972981

[ref9] Cook T. R.; Zheng Y.; Stang P. J. Metal–Organic Frameworks and Self-Assembled Supramolecular Coordination Complexes: Comparing and Contrasting the Design, Synthesis, and Functionality of Metal–Organic Materials. Chem. Rev. 2013, 113, 734–777. 10.1021/cr3002824. 23121121 PMC3764682

[ref10] Geng K.; He T.; Liu R.; Dalapati S.; Tan K. T.; Li Z.; Tao S.; Gong Y.; Jiang Q.; Jiang D. Covalent Organic Frameworks: Design, Synthesis, and Functions. Chem. Rev. 2020, 120, 8814–8933. 10.1021/acs.chemrev.9b00550. 31967791

[ref11] Abuzeid H. R.; El-Mahdy A. F.; Kuo S. Covalent organic frameworks: Design principles, synthetic strategies, and diverse applications. Giant 2021, 6, 100054 10.1016/j.giant.2021.100054.

[ref12] Usman M.; Mendiratta S.; Lu K. Semiconductor Metal-Organic Frameworks: Future Low-Bandgap Materials. Adv. Mater. 2017, 29, 1605071 10.1002/adma.201605071. 27859732

[ref13] Kolobov N.; Goesten M. G.; Gascon J. Metal–Organic Frameworks: Molecules or Semiconductors in Photocatalysis?. Angew. Chemie Int. Ed. 2021, 60, 26038–26052. 10.1002/anie.202106342. 34213064

[ref14] Zhang Y.; Liu H.; Gao F.; Tan X.; Cai Y.; Hu B.; Huang Q.; Fang M.; Wang X. Application of MOFs and COFs for photocatalysis in CO_2_ reduction, H_2_ generation, and environmental treatment. EnergyChem 2022, 4, 100078 10.1016/j.enchem.2022.100078.

[ref15] Kreno L. E.; Leong K.; Farha O. K.; Allendorf M.; Van Duyne R. P.; Hupp J. T. Metal–Organic Framework Materials as Chemical Sensors. Chem. Rev. 2012, 112, 1105–1125. 10.1021/cr200324t. 22070233

[ref16] Sun Y.; Zheng L.; Yang Y.; Qian X.; Fu T.; Li X.; Yang Z.; Yan H.; Cui C.; Tan W. Metal–Organic Framework Nanocarriers for Drug Delivery in Biomedical Applications. Nanomicro. Lett. 2020, 12, 103 10.1007/s40820-020-00423-3. 34138099 PMC7770922

[ref17] Bureekaew S.; Amirjalayer S.; Tafipolsky M.; Spickermann C.; Roy T. K.; Schmid R. MOF-FF - A flexible first-principles derived force field for metal-organic frameworks. Phys. Status Solidi B 2013, 250, 1128–1141. 10.1002/pssb.201248460.

[ref18] Boyd P. G.; Moosavi S. M.; Witman M.; Smit B. Force-Field Prediction of Materials Properties in Metal-Organic Frameworks. J. Phys. Chem. Lett. 2017, 8, 357–363. 10.1021/acs.jpclett.6b02532. 28008758 PMC5253710

[ref19] Chen T.; Manz T. A. A collection of forcefield precursors for metal–organic frameworks. RSC Adv. 2019, 9, 36492–36507. 10.1039/C9RA07327B. 35539031 PMC9075174

[ref20] Lin L.; Lee K.; Gagliardi L.; Neaton J. B.; Smit B. Force-Field Development from Electronic Structure Calculations with Periodic Boundary Conditions: Applications to Gaseous Adsorption and Transport in Metal–Organic Frameworks. J. Chem. Theory Comput. 2014, 10, 1477–1488. 10.1021/ct500094w. 26580364

[ref21] Bristow J. K.; Tiana D.; Walsh A. Transferable Force Field for Metal–Organic Frameworks from First-Principles: BTW-FF. J. Chem. Theory Comput. 2014, 10, 4644–4652. 10.1021/ct500515h. 25574157 PMC4284133

[ref22] Forrest K. A.; Pham T.; McLaughlin K.; Belof J. L.; Stern A. C.; Zaworotko M. J.; Space B. Simulation of the Mechanism of Gas Sorption in a Metal-Organic Framework with Open Metal Sites: Molecular Hydrogen in PCN-61. J. Phys. Chem. C 2012, 116, 15538–15549. 10.1021/jp306084t.

[ref23] Mercado R.; Vlaisavljevich B.; Lin L.; Lee K.; Lee Y.; Mason J. A.; Xiao D. J.; Gonzalez M. I.; Kapelewski M. T.; Neaton J. B.; Smit B. Force Field Development from Periodic Density Functional Theory Calculations for Gas Separation Applications Using Metal-Organic Frameworks. J. Phys. Chem. C 2016, 120, 12590–12604. 10.1021/acs.jpcc.6b03393.

[ref24] Becker T. M. Molecular Simulations of Tunable Materials. Ph.D. thesis, Delft University of Technology, 2019.

[ref25] Helgaker T.; Jørgensen P.; Olsen J. Molecular Electronic-Structure Theory; Wiley, 2000.

[ref26] Sholl D. S.; Steckel J. A. Density Functional Theory - a practical introduction; Wiley: Hoboken, NJ, 2009.

[ref27] Tuckerman M. E. Statistical Mechanics: Theory and Molecular Simulation; Oxford University Press: New York, 2010.

[ref28] Christensen A. S.; Kubař T.; Cui Q.; Elstner M. Semiempirical Quantum Mechanical Methods for Noncovalent Interactions for Chemical and Biochemical Applications. Chem. Rev. 2016, 116, 5301 10.1021/acs.chemrev.5b00584. 27074247 PMC4867870

[ref29] Elstner M.; Porezag D.; Jungnickel G.; Elsner J.; Haugk M.; Frauenheim T.; Suhai S.; Seifert G. Self-consistent-charge density-functional tight-binding method for simulations of complex materials properties. Phys. Rev. B 1998, 58, 7260–7268. 10.1103/PhysRevB.58.7260.

[ref30] Leong K.; Foster M. E.; Wong B. M.; Spoerke E. D.; Van Gough D.; Deaton J. C.; Allendorf M. D. Energy and charge transfer by donor–acceptor pairs confined in a metal–organic framework: a spectroscopic and computational investigation. J. Mater. Chem. A 2014, 2, 3389–3398. 10.1039/C3TA14328G.

[ref31] Li J.; Foster M. E.; Sohlberg K. Density-functional based tight-binding for the study of CO_2_/MOF interactions: the case of Zn(ADC)·DMSO. Mol. Simul. 2017, 43, 428–438. 10.1080/08927022.2016.1277024.

[ref32] Grimme S.; Antony J.; Ehrlich S.; Krieg H. A consistent and accurate ab initio parametrization of density functional dispersion correction (DFT-D) for the 94 elements H-Pu. J. Chem. Phys. 2010, 132, 154104 10.1063/1.3382344. 20423165

[ref33] Gaus M.; Cui Q.; Elstner M. DFTB3: Extension of the Self-Consistent-Charge Density-Functional Tight-Binding Method (SCC-DFTB). J. Chem. Theory Comput. 2011, 7, 931–948. 10.1021/ct100684s. PMC3509502 23204947

[ref34] Gaus M.; Goez A.; Elstner M. Parametrization and Benchmark of DFTB3 for Organic Molecules. J. Comput. Chem. 2013, 9, 338–354. 10.1021/ct300849w. 26589037

[ref35] Hofer T. S.; de Visser S. P. Quantum Mechanical/Molecular Mechanical Approaches for the Investigation of Chemical Systems – Recent Developments and Advanced Applications. Front. Chem. 2018, 6, 357 10.3389/fchem.2018.00357. 30271768 PMC6146044

[ref36] Li Y.; Wang X.; Xu D.; Chung J. D.; Kaviany M.; Huang B. H_2_O Adsorption/Desorption in MOF-74: Ab Initio Molecular Dynamics and Experiments. J. Phys. Chem. C 2015, 119, 13021–13031. 10.1021/acs.jpcc.5b02069.

[ref37] Rödl M.; Kerschbaumer S.; Kopacka H.; Blaser L.; Purtscher F. R. S.; Huppertz H.; Hofer T. S.; Schwartz H. A. Structural, dynamical, and photochemical properties of ortho-tetrafluoroazobenzene inside a flexible MOF under visible light irradiation. RSC Adv. 2021, 11, 3917–3930. 10.1039/D0RA10500G. 35424349 PMC8694203

[ref38] Rödl M.; Reka A.; Panic M.; Fischereder A.; Oberlechner M.; Mairegger T.; Kopacka H.; Huppertz H.; Hofer T. S.; Schwartz H. A. Fundamental Study of the Optical and Vibrational Properties of Fx-AZB@MOF systems as Functions of Dye Substitution and the Loading Amount. Langmuir 2022, 38, 4295–4309. 10.1021/acs.langmuir.1c03482. 35344366 PMC9009183

[ref39] Purtscher F. R. S.; Christanell L.; Schulte M.; Seiwald S.; Rödl M.; Ober I.; Maruschka L. K.; Khoder H.; Schwartz H. A.; Bendeif E.; Hofer T. S. Structural Properties of Metal–Organic Frameworks at Elevated Thermal Conditions via a Combined Density Functional Tight Binding Molecular Dynamics (DFTB MD) Approach. J Phys. Chem. C 2023, 127, 1560–1575. 10.1021/acs.jpcc.2c05103. PMC9884096 36721770

[ref40] Farrusseng D.; Daniel C.; Gaudillère C.; Ravon U.; Schuurman Y.; Mirodatos C.; Dubbeldam D.; Frost H.; Snurr R. Q. Heats of Adsorption for Seven Gases in Three Metal-Organic Frameworks: Systematic Comparison of Experiment and Simulation. Langmuir 2009, 25, 7383–7388. 10.1021/la900283t. 19496548

[ref41] Pianwanit A.; Kritayakornupong C.; Vongachariya A.; Selphusit N.; Ploymeerusmee T.; Remsungnen T.; Nuntasri D.; Fritzsche S.; Hannongbua S. The optimal binding sites of CH_4_ and CO_2_ molecules on the metal-organic framework MOF-5: ONIOM calculations. Chem. Phys. 2008, 349, 77–82. 10.1016/j.chemphys.2008.02.039.

[ref42] Einstein A. Über die von der molekularkinetischen Theorie der Wärme geforderte Bewegung von in ruhenden Flüssigkeiten suspendierten Teilchen. Ann. Phys. 1905, 322, 549–560. 10.1002/andp.19053220806.

[ref43] Babarao R.; Jiang J. Diffusion and Separation of CO_2_ and CH_4_ in Silicalite, C_168_ Schwarzite, and IRMOF-1: A Comparative Study from Molecular Dynamics Simulation. Langmuir 2008, 24, 5474–5484. 10.1021/la703434s. 18433152

[ref44] Keskin S. Molecular Dynamics - Theoretical Developments and Applications in Nanotechnology and Energy; InTech, 2012.

[ref45] Hofer T. S.; Kilchert F. M.; Tanjung B. A. An effective partial charge model for bulk and surface properties of cubic ZrO_2_, Y_2_O_3_ and yttrium-stabilised zirconia. Phys. Chem. Chem. Phys. 2019, 21, 25635–25648. 10.1039/C9CP04307A. 31720638

[ref46] Zhao Z.; Li Z.; Lin Y. S. Adsorption and Diffusion of Carbon Dioxide on Metal-Organic Framework (MOF-5). Ind. Eng. Chem. Res. 2009, 48, 10015–10020. 10.1021/ie900665f.

[ref47] Castellanos S.; Kapteijn F.; Gascon J. Photoswitchable metal organic frameworks: turn on the lights and close the windows. CrystEngComm 2016, 18, 4006–4012. 10.1039/C5CE02543E.

[ref48] Haldar R.; Heinke L.; Wöll C. Advanced Photoresponsive Materials Using the Metal–Organic Framework Approach. Adv. Mater. 2020, 32, 1905227 10.1002/adma.201905227. 31763731

[ref49] Huang Q.; Wu C. Photoswitching metal organic frameworks development and applications on environmental related topics. Mater. Today Sustain. 2022, 18, 100149 10.1016/j.mtsust.2022.100149.

[ref50] Schuster D.; Laggner C.; Langer T. Antitargets; John Wiley & Sons, Ltd, 2008; Chapter 1, pp 1–22.

[ref51] Xu Y.; Zhou Y.; Yu M.; Xiong Y.; Liu X.; Zhao Z. Excellent quantum yield enhancement in luminescent metal-organic layer for sensitive detection of antibiotics in aqueous medium. Dyes Pigm. 2022, 198, 109961 10.1016/j.dyepig.2021.109961.

[ref52] Liédana N.; Galve A.; Rubio C.; Téllez C.; Coronas J. CAF@ZIF-8: One-Step Encapsulation of Caffeine in MOF. ACS Appl. Mater. Interfaces 2012, 4, 5016–5021. 10.1021/am301365h. 22834763

[ref53] Javanbakht S.; Hemmati A.; Namazi H.; Heydari A. Carboxymethylcellulose-coated 5-fluorouracil@MOF-5 nano-hybrid as a bio-nanocomposite carrier for the anticancer oral delivery. Int. J. Biol. Macromol. 2020, 155, 876–882. 10.1016/j.ijbiomac.2019.12.007. 31805324

[ref54] Tanaka S.; Tanaka Y. A Simple Step toward Enhancing Hydrothermal Stability of ZIF-8. ACS Omega 2019, 4, 19905–19912. 10.1021/acsomega.9b02812. 31788623 PMC6882103

[ref55] Goyal P.; Qian H.; Irle S.; Lu X.; Roston D.; Mori T.; Elstner M.; Cui Q. Molecular Simulation of Water and Hydration Effects in Different Environments: Challenges and Developments for DFTB Based Models. J. Phys. Chem. B 2014, 118, 11007–11027. 10.1021/jp503372v. 25166899 PMC4174991

[ref56] Devereux C.; Smith J. S.; Huddleston K. K.; Barros K.; Zubatyuk R.; Isayev O.; Roitberg A. E. Extending the Applicability of the ANI Deep Learning Molecular Potential to Sulfur and Halogens. J. Chem. Theory Comput. 2020, 16, 4192–4202. 10.1021/acs.jctc.0c00121. 32543858

[ref57] Kriesche B. M.; Kronenberg L. E.; Purtscher F. R. S.; Hofer T. S. Storage and diffusion of CO_2_ in covalent organic frameworks—A neural network-based molecular dynamics simulation approach. Front. Chem. 2023, 11, 1100210 10.3389/fchem.2023.1100210. 36970402 PMC10033539

