# Supplementary material for: Beyond the Status Quo: Density Functional Tight Binding and Neural Network Potentials as a Versatile Simulation Strategy to Characterize Host–Guest Interactions in Metal- and Covalent Organic Frameworks
Source: J Phys Chem Lett. 2023 Jun 23;14(26):6018–27. doi: 10.1021/acs.jpclett.3c00941 (PMC10331828; doi:10.1021/acs.jpclett.3c00941)
Supplement: Supplementary file 1 — jz3c00941_si_001.pdf [file jz3c00941_si_001.pdf]

Beyond the Status Quo: Density Functional Tight Binding and Neural Network Potentials as a Versatile Simulation Strategy to Characterise Host-Guest interactions in Metal- and Covalent Organic Frameworks

Supplementary Material

Thomas S. Hofer[*a], Risnita Vicky Listyarini[a], Emir Hajdarevic[a,b], Lukas Maier[a], Felix R. S. Purtscher[a], Jakob Gamper[a], Friedrich Hanser[b]*

[a] Institute of General, Inorganic and Theoretical Chemistry
Center for Chemistry and Biomedicine
University of Innsbruck, Innrain 80-82, A-6020 Innsbruck, Austria

[b]Institute of Electrical and Biomedical Engineering
UMIT Tirol, Eduard-Wallnöfer-Zentrum 1
A-6060 Hall in Tirol, Austria

E-Mail: T.Hofer@uibk.ac.at
Tel.: +43-512-507-57111
Fax: +43-512-507-57199

June 22, 2023

[*]Corresponding author

S1 Methods

In the following details of the employed SCC DFTB/3ob/D3 MD protocol are provided. In addition, the applied analysis methods are outlined and details on the quantum chemical calculation of the indigo molecule are provided.

S1.1 SCC DFTB MD simulations

S1.1.1 SCC DFTB

Following the successful examples of previous investigations focused on fluorinated azobenzenes embedded in DMOF-1,[1,2] the self-consistent charge density functional tight binding (SCC DFTB)[3–5] approach was applied in combination with the 3ob parameter set[6,7] using the DFTB+ package.[8] Due to the comparably large cell parameters of the investigated MOF systems so-called Γ-point sampling of the Brillouin zone is adequate to model the intermolecular forces as shown previously.[9] A damping factor $\zeta = 4.0$ is applied to downscale all interactions involving hydrogen atoms as required by the 3ob parametrisation.[6] To improve the description of dispersive contributions the Grimme D3 correction employing Becke-Johnson damping[10,11] was applied. The convergence criterion for the SCC DFTB iterations was set to $\leq 10^{-6}$ Hartree corresponding to a convergence in the SCC error of approx. 10^{-4}.

S1.1.2 Molecular dynamics

The DFTB+ package has been interfaced to our in-house QM/MM MD simulation program[12–15] to execute the individual steps of the molecular dynamics simulation. To facilitate an MD timestep of 2.0fs, the velocity-Verlet time integrator[16] in conjunction with the SHAKE/RATTLE algorithms[16] has been applied to satisfy holonomic constraints applied to all hydrogen-containing bonds. The simulations were carried out in the isothermal–isobaric (NPT) ensemble utilising

a Nosé-Hoover chain thermostat[17] with the respective chain length set to 5. The Berendsen manostat algorithm[18] was employed to maintain constant pressure throughout the simulations, the corresponding relaxation parameter was set to 10 ps. Generation of MD snapshots have been carried out using the VMD (visual molecular dynamics) program.[19]

All simulations of the pristine MOFs have been started from an optimized structure obtained *via* structure optimization carried out via DFTB+. After an initial pre-equilibration to the target temperature, the systems have been equilibrated for at least 5000 MD steps (10 ps) followed by sampling times in the range from 125 000 to 250 000 MD steps (250 to 500 ps).

Guest molecules have been inserted into the equilibrated host systems at random, ensuring a minimum distance of 1.5 Å between the respective atoms. The same equilibration protocol as in case of the pristine systems has been applied. In investigations of the diffusion coefficient a minimum simulation time of 0.5 ns was applied for each studied temperature following an extended equilibration period of at least 25ps.

S1.2 Quantum chemical calculation of indigo

The dipole moments of the E- and Z-from of the indigo molecule (2,2′-Bis(2,3-dihydro-3-oxoindolyliden) in the respective minimum configuration have been evaluated based on B3LYP[20] and MP2[21] (Møller-Plesset perturbation theory of second order) calculations employing the 6-311G(d,p) basis set[22] (obtained *via* the Basis Set Exchange website[23,24]) using Gaussian16.[25] In case of the B3LYP calculation again the respective Grimme D3 dispersion correction based on Becke-Johnson damping[10,11] has been applied.

S1.3 Analysis

S1.3.1 Determination of powder X-ray diffraction patterns

A comparison between the structural description obtained *via* energy minimisations (including also the cell parameters in the optimization) as well as in the MD simulations with experimental reference data obtained at Mo K_α radiation (λ =0.709319 nm) has been carried out in case of MOF-5 and ZIF-8.[9] The respective powder X-ray diffraction (PXRDs) patterns have been determined employing the RIETAN-FP program[26] as available in the command line interface of VESTA.[27] In case of the MD simulations, a total of 5 000 individual PXRD patterns obtained for snapshots taken at regular intervals of 20 fs over an equilibrated simulation trajectory of 100 ps have been calculated and subsequently averaged.

S1.3.2 Determination of the linear thermal expansion coefficient

Based on the simulation data the linear thermal expansion coefficient α_a can be determined *via*

$$\alpha_a^{298\mathrm{K}} = \frac{1}{\langle a^{298\mathrm{K}} \rangle} \frac{\partial a}{\partial T} \tag{S1}$$

with the respective slope being determined via finite differentiation employing a total of four neighbouring temperature points:

$$\frac{\partial a}{\partial T} \approx \frac{\langle a^{248\mathrm{K}} \rangle - 8 \langle a^{273\mathrm{K}} \rangle + 8 \langle a^{323\mathrm{K}} \rangle - \langle a^{348\mathrm{K}} \rangle}{12 \Delta T} \tag{S2}$$

Following an initial equilibration at the target temperature for a minimum of 10 000 MD steps (20 ps), another 25 000 MD steps (50 ps) have been employed to ensure proper equilibration under NPT conditions. Next, a minimum of 125 000 MD steps (0.25 ns) have been employed to determine the average lattice parameter $\langle a^T \rangle$ at the respective temperature T.

S1.3.3 Evaluation of the interaction energy

The interaction energies U_{int} between the MOF-5 host and the guest molecules CO_2 were determined according to

$$U_{\text{int}} = U_{\text{guest@MOF}} - \langle U_{\text{MOF}} \rangle - \langle U_{\text{guest}} \rangle \tag{S3}$$

with $U_{\text{guest@MOF}}$ being the total energy (*i.e.* kinetic plus potential energy) of the combined guest@host system. U_{MOF} and U_{guest} correspond to the respective total energies obtained for the isolated compounds employing the same simulation setup. In case of the indigo guest molecules, both the E- and Z-conformer have been considered in separate simulations. Since in this case the molecule is treated in absence of the MOF host (*i.e. in vacuo* conditions), pressure coupling was not applied. In all simulations of the isolated systems at least 10 ps of equilibration (5 000 MD steps) have been performed followed by 100 ps of sampling (50 000 MD steps). The respective ensemble averages denoted as $\langle \ldots \rangle$ were then determined over the last 50 ps of the simulation trajectory.

S1.3.4 Characterisation of CO_2 diffusion in MOF-5

The self-diffusion coefficient D_s of carbon dioxide in MOF-5 was evaluated via the Einstein relation[28] given as

$$D_s = \frac{1}{2d} \lim_{t \to \infty} \frac{\left\langle (\mathbf{r}_t - \mathbf{r}_0)^2 \right\rangle}{t} \tag{S4}$$

with d being the dimensionality of the system, $\mathbf{r}_t$ and $\mathbf{r}_0$ correspond to the position of the carbon atom of CO_2 at a given time origin and time t, respectively.

A running correlation window of 5 ps (1250 MD configurations) proved adequate in the determination of the diffusion coefficient, if only the last 625 points of the correlation interval

(*i.e.* 2.5 ps) were employed in the associated linear fit. For each of the seven considered temperatures in the range from 248.15 to 398.15 K a total sampling period of $\geq$0.5 ns ($\geq$250 000 MD steps) was considered, to ensure that the simulation time is significantly larger than the correlation length.

Figure S02 depicts the respective mean square displacements (MSDs) for the seven considered temperatures over the correlation window of 5ps. In all cases a near-perfect linearity of the MSD is observed in the long-time limit. Quadratic fits applied to the first 0.1 ps (*i.e.* the first 26 datapoints) clearly highlight that the diffusive region is well separated from the respective ballistic regime.

The associated activation energy E_a can then be determined via a linear fit to the respective Arrhenius representation given as

$$\ln(D_s) = \ln(D_0) - \frac{E_a}{R}\frac{1}{T} \tag{S5}$$

with D_0 being the associated pre-exponential factor and R corresponds to the molar gas constant.

Typically, at higher simulation temperatures more diffusive events are observed, making estimates for D_s more reliable at elevated thermal conditions. For this reason, the linear fit to determine the activation energy has been carried out employing *i*) all seven simulation temperatures as well as *ii*) considering only the four highest temperatures in the set.

S2 Thermal expansion coefficients of MOF systems

MOF-5	SCC DFTB/3ob/D3 MD	-12.7	this work
	PND	-16 to -10	[29]
	PND	-12.0	[30]
	PXRD	-13.1	[31]
	PXRD	-14.5	[30]
	MM MD	–5.27	[32]
	MM MD	-8.0*	[33]
	MM MD	-10.1*	[34]
	MM MD	-12.3*	[35]
	MM MD	-18.3*	[36]
	MM MD	-26.2 to -12	[37]
	MM PIMD	-13.7*	[38]
	MM MD / PIMD (harmonic)	-21.5 to -19.1	[39]
	MM MD / PIMD (anharmonic)	-16.4 to -14.2	[39]
	QHA LD (B3LYP-D3)	-10.6	[40]
ZIF-8	SCC DFTB/3ob/D3 MD	14.6	this work
	PXRD	11.9	[41]
	PXRD	6.5*	[42]
UMCM-9	SCC DFTB/3ob/D3 MD	-9.5	this work
	MM MD	-12.5*	[35]

* derived from the volumetric thermal expansion via $\alpha_a = \frac{\alpha_V}{3}$

Table S1: Comparison of the linear thermal expansion coefficient α_a in MK^{-1} obtained from the SCC DFTB/3ob/D3 MD simulation protocol with data reported in the literature. (PND powder neutron diffraction; PXRD powder X-ray diffraction; MM molecular mechanics; MD molecular dynamics, PIMD path-integral molecular dynamics; QHA LD quasi-harmonic approximation lattice dynamics)

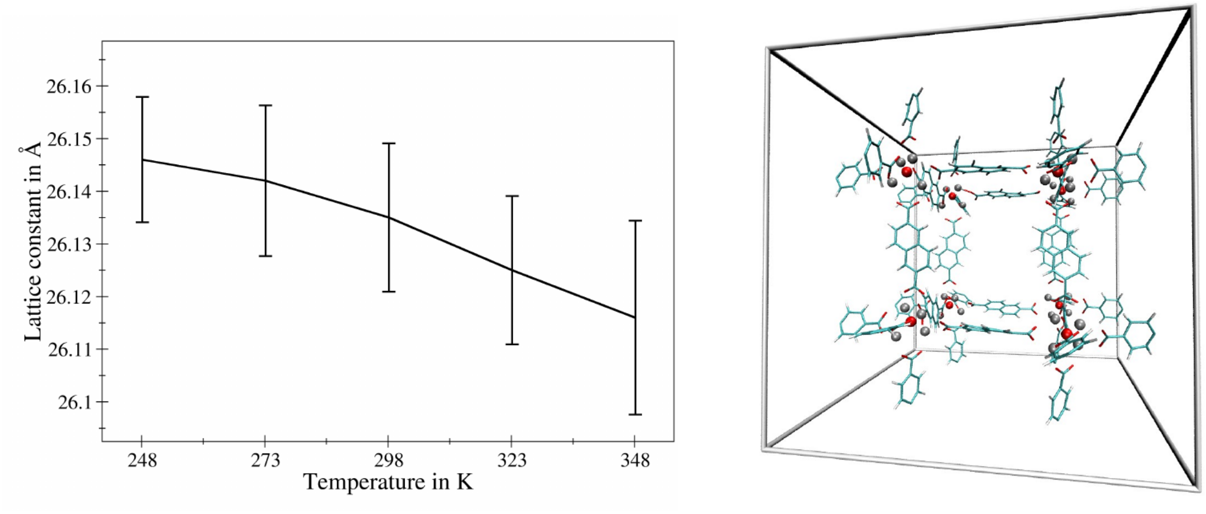

Figure S1: Left: Temperature dependence of the lattice constant of UMCM-9 determined *via* SCC DFTB/3ob/D3 MD simulations displaying a negative thermal expansion. Right: Unit cell of the UMCM-9 simulation system.

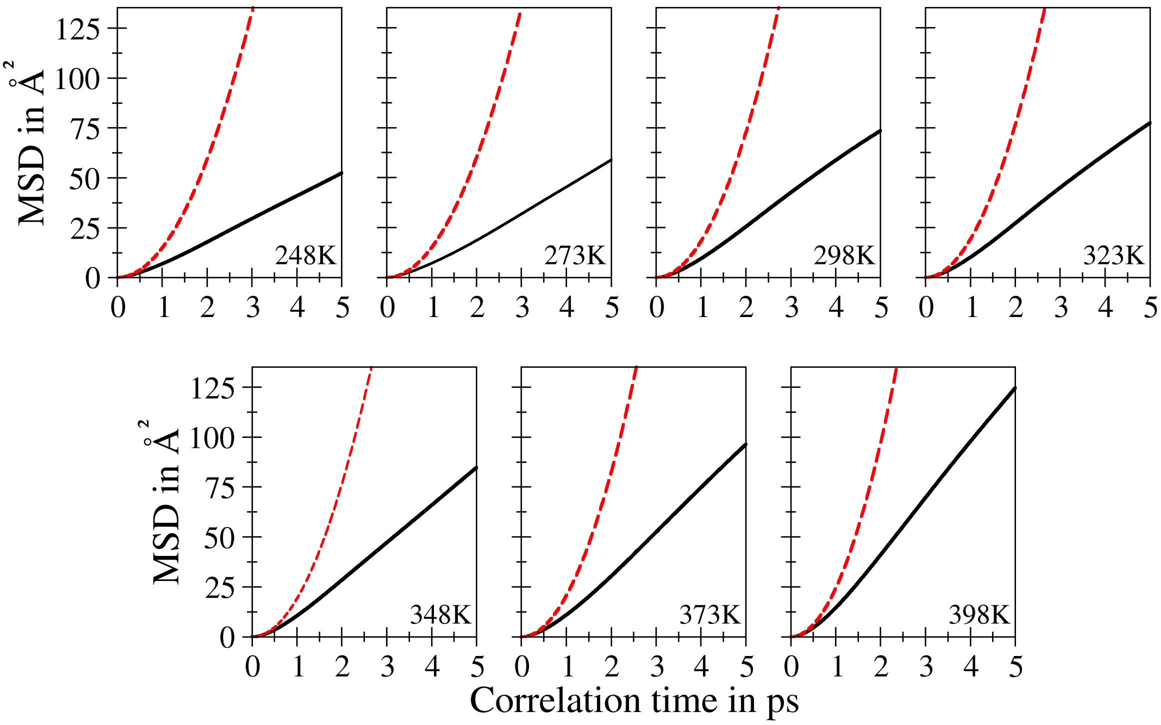

Figure S2: Mean square displacement (MSD, black) determined for CO_2@MOF-5 in the temperature range of 248 to 398K employing a correlation window of 5ps. In all cases textbook-like behaviour of the MSD is observed. In order to highlight, that for all temperatures the long-time limit has been reached, a square function has been fitted to the first 0.1ps (first 26 data points) of the correlation windows (red) to depict the corresponding ballistic regime. Only the range from 2.5 to 5.0 ps has been considered in the linear fit to determine the diffusion coefficient shown in figure 2c of the main manuscript.

References

(1) Rödl, M.; Kerschbaumer, S.; Kopacka, H.; Blaser, L.; Purtscher, F. R. S.; Huppertz, H.; Hofer, T. S.; Schwartz, H. A. Structural, dynamical, and photochemical properties of ortho-tetrafluoroazobenzene inside a flexible MOF under visible light irradiation. *RSC Adv.* **2021**, *11*, 3917 – 3930.

(2) Rödl, M.; Reka, A.; Panic, M.; Fischereder, A.; Oberlechner, M.; Mairegger, T.; Kopacka, H.; Huppertz, H.; Hofer, T. S.; Schwartz, H. A. Fundamental Study of the Optical and Vibrational Properties of Fx-AZB@MOF systems as Functions of Dye Substitution and the Loading Amount. *Langmuir* **2022**, *38*, 4295 – 4309.

(3) Elstner, M.; Porezag, D.; Jungnickel, G.; Elsner, J.; Haugk, M.; Frauenheim, T.; Suhai, S.; Seifert, G. Self-consistent-charge density-functional tight-binding method for simulations of complex materials properties. *Phys. Rev. B* **1998**, *58*, 7260 – 7268.

(4) Oliveira, A. F.; Seifert, G.; Heine, T.; Duarte, H. A. Density-functional based tight-binding: an approximate DFT method. *J. Braz. Chem. Soc.* **2009**, *20*, 1193.

(5) Spiegelman, F.; Tarrat, N.; Cuny, J.; Dontot, L.; Posenitskiy, E.; Martí, C.; Simon, A.; Rapacioli, M. Density-functional tight-binding: basic concepts and applications to molecules and clusters. *Adv. Phys. X* **2020**, *5*, 1710252.

(6) Gaus, M.; Goez, A.; Elstner, M. Parametrization and Benchmark of DFTB3 for Organic Molecules. *J. Comput. Chem.* **2013**, *9*, 338 – 354.

(7) Lu, X.; Gaus, M.; Elstner, M.; Cui, Q. Parametrization of DFTB3/3OB for Magnesium and Zinc for Chemical and Biological Applications. *J. Phys. Chem. B* **2015**, *119*, 1062 – 1082.

(8) Hourahine, B.; Aradi, B.; Blum, V.; Bonafé, F.; Buccheri, A.; Camacho, C.; Cevallos, C.; Deshaye, M. Y.; Dumitrică, T.; Dominguez, A.; Ehlert, S.; Elstner, M.; van der Heide, T.; Hermann, J.; Irle, S.; Kranz, J. J.; Köhler, C.; Kowalczyk, T.; Kubař, T.; Lee, I. S.; Lutsker, V.; Maurer, R. J.; Min, S. K.; Mitchell, I.; Negre, C.; Niehaus, T. A.; Niklasson, A. M. N.; Page, A. J.; Pecchia, A.; Penazzi, G.; Persson, M. P.; Řezáč, J.; Sánchez, C. G.; Sternberg, M.; Stöhr, M.; Stuckenberg, F.; Tkatchenko, A.; Yu, V. W.; Frauenheim, T. DFTB+, a software package for efficient approximate density functional theory based atomistic simulations. *J. Chem. Phys.* **2020**, *152*, 124101.

(9) Purtscher, F. R. S.; Christanell, L.; Schulte, M.; Seiwald, S.; Rödl, M.; Ober, I.; Maruschka, L. K.; Khoder, H.; Schwartz, H. A.; Bendeif, E.; Hofer, T. S. Structural Properties of Metal–Organic Frameworks at Elevated Thermal Conditions via a Combined Density Functional Tight Binding Molecular Dynamics (DFTB MD) Approach. *J Phys. Chem. C* **2023**, *127*, 1560 – 1575.

(10) Grimme, S.; Antony, J.; Ehrlich, S.; Krieg, H. A consistent and accurate ab initio parametrization of density functional dispersion correction (DFT-D) for the 94 elements H-Pu. *J. Chem. Phys.* **2010**, *132*, 154104.

(11) Grimme, S.; Ehrlich, S.; Goerigk, L. Effect of the damping function in dispersion corrected density functional theory. *J. Comput. Chem.* **2011**, *32*, 1456 – 1465.

(12) Hofer, T. S.; Tirler, A. O. Combining 2d-Periodic Quantum Chemistry with Molecular Force Fields: A Novel QM/MM Procedure for the Treatment of Solid-State Surfaces and Interfaces. *J. Chem. Theory Comput.* **2015**, *11*.

(13) Saleh, M.; Hofer, T. S. A DFTB/MM MD Approach for Solid-State Interfaces: Structural

and Dynamical Properties of H_2O and NH_3 on R-TiO_2(001). *J. Phys. Chem. C* **2019**, *123*, 7230 – 7245.

(14) Prasetyo, N.; Hofer, T. S. Adsorption and dissociation of water molecules at the α-Al_2O_3(0001) surface: A 2-dimensional hybrid self-consistent charge density functional based tight-binding/molecular mechanics molecular dynamics (2D SCC-DFTB/MM MD) simulation study. *Comp. Mat. Sci.* **2019**, *164*, 195 – 204.

(15) Kriesche, B. M.; Kronenberg, L. E.; Purtscher, F. R. S.; Hofer, T. S. Storage and diffusion of CO_2 in covalent organic frameworks—A neural network-based molecular dynamics simulation approach. *Front. Chem.* **2023**, *11*, 1100210.

(16) Tuckerman, M. E. *Statistical Mechanics: Theory and Molecular Simulation*; Oxford University Press: New York, 2010.

(17) Martyna, G. J.; Klein, M. L. Nose-Hoover chains: The canonical ensemble via continuos dynamics. *J. Chem. Phys.* **1992**, *97*, 2635 – 2645.

(18) Berendsen, H. J. C.; Postma, J. P. M.; van Gunsteren, W. F.; DiNola, A.; Haak, J. R. Molecular Dynamics with coupling to an external bath. *J. Chem. Phys.* **1984**, *81*, 3684 – 3690.

(19) Humphrey, W.; Dalke, A.; Schulten, K. VMD - Visual Molecular Dynamics. *J. Mol. Graphics* **1996**, *14*, 33 – 38.

(20) Becke, A. D. Density-functional thermochemistry. III. The role of exact exchange. *J. Chem. Phys.* **1993**, *98*, 5648 – 5652.

(21) Møller, C.; Plesset, M. S. Note on an Approximation Treatment for Many-Electron Systems. *Phys. Rev.* **1934**, *46*, 618 – 622.

(22) Krishnan, R.; Binkley, J. S.; Seeger, R.; Pople, J. A. Self-consistent molecular orbital methods. XX. A basis set for correlated wave functions. *J. Chem. Phys.* **1980**, *72*, 650 – 654.

(23) Schuchardt, K. L.; Didier, B. T.; Elsethagen, T.; Sun, L.; Gurumoorthi, V.; Chase, J.; Li, J.; Windus, T. L. Basis Set Exchange: A Community Database for Computational Sciences. *J. Chem. Inf. Model.* **2007**, *47*, 1045 – 1052.

(24) Pritchard., B. P.; Altarawy, D.; Didier, B.; Gibsom, T. D.; Windus, T. L. A New Basis Set Exchange: An Open, Up-to-date Resource for the Molecular Sciences Community. *J. Chem. Inf. Model.* **2019**, *59*, 4814 – 4820.

(25) Frisch, M. J.; Trucks, G. W.; Schlegel, H. B.; Scuseria, G. E.; Robb, M. A.; Cheeseman, J. R.; Scalmani, G.; Barone, V.; Petersson, G. A.; Nakatsuji, H.; Li, X.; Caricato, M.; Marenich, A. V.; Bloino, J.; Janesko, B. G.; Gomperts, R.; Mennucci, B.; Hratchian, H. P.; Ortiz, J. V.; Izmaylov, A. F.; Sonnenberg, J. L.; Williams-Young, D.; Ding, F.; Lipparini, F.; Egidi, F.; Goings, J.; Peng, B.; Petrone, A.; Henderson, T.; Ranasinghe, D.; Zakrzewski, V. G.; Gao, J.; Rega, N.; Zheng, G.; Liang, W.; Hada, M.; Ehara, M.; Toyota, K.; Fukuda, R.; Hasegawa, J.; Ishida, M.; Nakajima, T.; Honda, Y.; Kitao, O.; Nakai, H.; Vreven, T.; Throssell, K.; Montgomery, J. A., Jr.; Peralta, J. E.; Ogliaro, F.; Bearpark, M. J.; Heyd, J. J.; Brothers, E. N.; Kudin, K. N.; Staroverov, V. N.; Keith, T. A.; Kobayashi, R.; Normand, J.; Raghavachari, K.; Rendell, A. P.; Burant, J. C.; Iyengar, S. S.; Tomasi, J.; Cossi, M.; Millam, J. M.; Klene, M.; Adamo, C.; Cammi, R.; Ochterski, J. W.; Martin, R. L.; Morokuma, K.; Farkas, O.; Foresman, J. B.; Fox, D. J. Gaussian˜16 Revision C.01. 2016; Gaussian Inc. Wallingford CT.

(26) Izumi, F.; Momma, K. Three-Dimensional Visualization in Powder Diffraction. *Solid State Phenom.* **2007**, *130*, 15 – 20.

(27) Momma, K.; Izumi, F. VESTA 3for three-dimensional visualization of crystal, volumetric and morphology data. *J. Appl. Crystallogr.* **2011**, *44*, 1272 – 1276.

(28) Einstein, A. Über die von der molekularkinetischen Theorie der Wärme geforderte Bewegung von in ruhenden Flüssigkeiten suspendierten Teilchen. *Ann. Phys.* **1905**, *322*, 549 – 560.

(29) Zhou, W.; Wu, H.; Yildirim, T.; Simpson, J. R.; Walker, A. R. H. Origin of the exceptional negative thermal expansion in metal-organic framework-5 Zn_4O(1,4-benzenedicarboxylate)$_3$. *Phys. Rev. B* **2008**, *78*, 054114.

(30) Lock, N.; Christensen, M.; Wu, Y.; Peterson, V. K.; Thomsen, M. K.; Piltz, R. O.; Ramirez-Cuesta, A. J.; McIntyre, G. J.; Norén, K.; Kutteh, R.; Kepert, C. J.; Kearley, G. J.; Iversen, B. B. Scrutinizing negative thermal expansion in MOF-5 by scattering techniques and ab initio calculations. *Dalton Trans.* **2013**, *42*, 1996 – 2007.

(31) Lock, N.; Wu, Y.; Christensen, M.; Cameron, L. J.; Peterson, V. K.; Bridgeman, A. J.; Kepert, C. J.; Iversen, B. B. Elucidating Negative Thermal Expansion in MOF-5. *J. Phys. Chem. C* **2010**, *114*, 16181–16186.

(32) Bristow, J. K.; Tiana, D.; Walsh, A. Transferable Force Field for Metal–Organic Frameworks from First-Principles: BTW-FF. *J. Chem. Theory Comput.* **2014**, *10*, 4644 – 4652.

(33) Han, S. S.; Goddard, W. A. Metal-Organic Frameworks Provide Large Negative Thermal Expansion Behavior. *J. Phys. Chem. C* **2007**, *111*, 15185 – 15191.

(34) Sun, Y.; Sun, H. An all-atom force field developed for $Zn_4O(RCO_2)_6$ metal organic frameworks. *J Mol. Model.* **2014**, *20*, 2146.

(35) Wieme, J.; Van Speybroeck, V. Unravelling thermal stress due to thermal expansion mis-

match in metal–organic frameworks for methane storage. *J. Mater. Chem. A* **2021**, *9*, 4898 – 4906.

(36) Dubbeldam, D.; Walton, K. S.; Ellis, D. E.; Snurr, R. Q. Exceptional Negative Thermal Expansion in Isoreticular Metal–Organic Frameworks. *Angew. Chem. Int. Ed.* **2007**, *46*, 4496 – 4499.

(37) Boyd, P. G.; Moosavi, S. M.; Witman, M.; Smit, B. Force-Field Prediction of Materials Properties in Metal-Organic Frameworks. *J. Phys. Chem. Lett.* **2017**, *8*, 357 – 363.

(38) Wieme, J.; Vandenbrande, S.; Lamaire, A.; Kapil, V.; Vanduyfhuys, L.; Van Speybroeck, V. Thermal Engineering of Metal–Organic Frameworks for Adsorption Applications: A Molecular Simulation Perspective. *ACS Appl. Mater. Interfaces* **2019**, *11*, 38697 – 38707.

(39) Lamaire, A.; Wieme, J.; Rogge, S. M. J.; Waroquier, M.; Van Speybroeck, V. On the importance of anharmonicities and nuclear quantum effects in modelling the structural properties and thermal expansion of MOF-5. *J. Chem. Phys.* **2019**, *150*, 094503.

(40) Ryder, M. R.; Maul, J.; Civalleri, B.; Erba, A. Quasi-Harmonic Lattice Dynamics of a Prototypical Metal–Organic Framework. *Adv. Theory Simul.* **2019**, *2*, 1900093.

(41) Sapnik, A. F.; Geddes, H. S.; Reynolds, E. M.; Yeung, H. H.; Goodwin, A. L. Compositional inhomogeneity and tuneable thermal expansion in mixed-metal ZIF-8 analogues. *Chem. Commun.* **2018**, *54*, 9651 – 9654.

(42) Burtch, N. C. *Engineering Precisely Controlled Negative and Zero Thermal Expansion Behaviors in Metal-Organic Frameworks.*; United States: 2019, Web DOI: 10.2172/1561441.
